# The role of innovative technology in multicultural vocational tourism education

**DOI:** 10.3389/fpsyg.2023.1091881

**Published:** 2023-02-15

**Authors:** Nesrin M. Bahçelerli

**Affiliations:** Faculty of Tourism, Near East University, Nicosia, Cyprus

**Keywords:** innovative technology, multicultural, vocational tourism education, multicultural classroom, student

## Introduction

Research has shown that the use of multimedia in the classroom improves creativity, innovative problem solving, and communication between people. Using technology allows educators to improve their teaching strategies and learning processes and to be more inclusive of all learning styles. Such advances in technology have enabled the development of vocational education and the sector seeks students for their technological competencies. International students have created a multicultural education environment in universities. In a multicultural learning environment, the use of innovative technologies is important, especially in vocational tourism education where applied education takes place. In this research, it is aimed to reveal the role of innovative technology in multicultural vocational tourism education. The study was carried out in accordance with action research, one of the qualitative research methods. The study population of the research is university students who take courses in a multicultural classroom. The study universe was made in accordance with the purposeful sampling technique. In the study, 36 students were interviewed. As a result of the research, they put forward the view that innovative technologies will benefit their professional lives. Students stated that especially in online education environments, inequality in multicultural classrooms and the different technology backgrounds of the students caused difficulties.

Today, while underdeveloped and developing countries are trying to catch up with developed countries, industrialized countries are striving for advanced technology and strong economies by controlling certain technologies. A sustainable and developed economy also requires an innovative approach (Schroeder et al., 2018). Because a growing and rapidly developing system requires a competent and equipped workforce.

Since the innovation process in cooperation with education in the global economy is accepted as an absolute prerequisite for the modern world, the innovation theory of education has emerged as a new research area in science pedagogy (Stukalenko et al., 2016). Innovation in education is an approach that avoids traditional practices and aims to create better learning outcomes for students using the same or less time and resources (Fayzievna et al., 2020). Innovation theory deals with the innovation processes of the education system. Innovation processes emerge through innovative learning activities, innovations and innovative learning environments (Kharatova and Ismailov, 2022). The changing student profile, the effects of new technologies and the changing demands of the business world also affect the education given in schools. In parallel with the applied education model, the education given in schools should support real life conditions and develop innovative skills (Atamanyuk et al., 2021; Maiorov, 2021).

Globalization has accelerated the acceleration of world trade, the emergence of multinational companies, the common policies of countries, fast and easy transportation, disasters such as poverty, environmental disasters, exploitation and terrorism. However, the acceleration of information flow with globalization shows that the new age is the information age and nations are seeking information to adapt to this age. Sending students abroad is seen as the first and most common method of educated workforce and knowledge transfer in developing countries. Because student mobility was seen as the shortest way to bring knowledge. International studentship is becoming more and more common around the world. In addition to acquiring knowledge, the focus is on getting to know different cultures, meeting new people, the ability to communicate between cultures and developing new ways of thinking. In addition, the opportunity to learn different languages, develop cultural competence, find a better job and achieve a higher status is seen as an advantage of studying abroad. For these reasons, it is becoming more and more common among young people to study in another country. Therefore, multicultural education environments are formed in higher education (Zimmermann et al., 2021).

Culture is defined as a versatile dynamic structure that exists in all areas of life and enables self-expression through teaching. This dynamic structure is not only complex but also interactive, enabling individuals and institutions to integrate into society (Jones et al., 2017). With the development of the world, universal cultures began to rise instead of local cultures. Since this process is also reflected in education, the concept of multicultural education has become more important. Each researcher has defined multicultural education from a different perspective. The definition accepted by most is a reform movement for all students to benefit equally from education and to create equal educational conditions for all students, regardless of race, culture, gender, belief, language and social class (Bilgin and Oksal, 2018).

Sociocultural differences arising from differences in multiple interdependent identity categories – social class, race, ethnicity, language, gender, disability, sexual orientation, age, and religion and inequalities in access and quality of education and social participation are the norm, rather than a myth or an error (Banks, 2013; Bista, 2022). Equality is a dynamic, dialogic and collective system of conditions-processes-goals that society achieves through its central mission to educate everyone, not just those with undeserved privilege (James, 2008; Ladson-Billings, 2021). Studies show that when multimedia is used in the classroom, creativity increases innovative problem-solving skills and improves interpersonal communication. The use of technology in education provides an opportunity for teachers to improve their teaching strategies and learning processes and to create a more comprehensive learning style (Haleem et al., 2022). Thanks to technology, the easy sharing of knowledge and experience effectively shrinks the world. Nowadays, it is easy to see in its multilingual and multicultural classrooms (Hutchison, 2021). With technology, students in a multicultural learning environment can find opportunities to gain class status more easily than in traditional competitive classrooms, as well as revealing their strengths. Collaborative learning or interactive learning environments are created in digital teaching environments so that students research and present solutions for their concerns (Haleem et al., 2022). Technology can simulate real-life environments and promotes learning by doing. Technology can help solve problems that arise in multicultural learning environments.

Tourism education meets the current needs of the travel and tourism industry in parallel with other vocational education fields (Chandra et al., 2022). In addition, special attention is paid to the optimization of learning processes. In this regard, special attention is paid to the implementation of innovative technologies that allow future employees of the tourism industry to acquire the necessary knowledge, expertise and skills using non-standard technologies (Shin and Perdue, 2022). Applying such technologies can create a link between theoretical training and the acquisition of hands-on skills, the application of new learning forms and methods, and the promotion of the use of individualized learning. It allows students to be educated according to today’s needs and requirements (Kallou and Kikilia, 2021).

Technological transformations in the production and service sectors have begun to change professional qualifications (Gronau et al., 2017) and necessitated the vocational education process to be addressed from this perspective as well. It is stated that the qualifications of the manpower and vocational and technical education have the power to greatly affect the industrial and economic development of the country. Technological development and innovation have always affected workflows and competencies needed for skilled jobs (Little et al., 2020).

In the current situation, there is a need for new techniques in tourism education, where online education is more important than ever. Among these, developmental learning, problem-based learning, multi-level learning, collective learning system, research problem solving technique, application of research method in teaching, project methods, discussion technology, modular and block modular technology come to the fore. In the literature, there are innovative educational techniques such as critical thinking development technology, technology of using game methods in education: role-playing games, business and other educational games, cooperative learning, information and communication technologies, health technologies, innovative assessment systems (Kazmina et al., 2020). Informative learning techniques, game learning techniques, interactive learning techniques, problematic learning techniques and project learning techniques are distinguished in terms of content features. These techniques are also valid in vocational education, which is the training of a specialist in a particular field who is oriented toward modern, economic and social reality and can do the best in the chosen profession. In this instance, it is necessary to focus on advanced technologies at the current level of development and technologies of the future, that is, developments in the tourism sector. Because after the end of the education, that is, with the acquisition of basic knowledge and skills, the student enters an active working life within a few years. Project technologies, brainstorming, problem solving, management decisions in the form of simulation, interactive and video technologies are important in modern professional tourism education (Allcoat and Von Muhlenen, 2018). Thus, it is foreseen that it will be possible to raise individuals with the necessary skills and abilities, which are the expectations of the sector.

In order to keep up with the high industrial and market demands, it is important for students and future professionals to adopt new technologies. These technologies not only prepare them for future markets and jobs, but also act as tools for better and faster learning by helping students better visualize new content (Chandra et al., 2022).

Due to the international nature of the tourism industry, students have international career opportunities. Therefore, in order to avoid difficult situations that may arise from the lack of cultural differences, the importance of studying in multicultural educational environments throughout their education is put forward (Suh et al., 2012; Nachmias et al., 2017). Vocational training programs in the tourism industry should provide future tourism leaders with the skills necessary to operate and advance in cultural diversity in an increasingly international industry (Grobelna, 2015).

The picture drawn with the pandemic reveals the importance and necessity of digital transformation in the entire education system in general and in the higher education system in particular (Bozkurt, 2020). However, digital transformation should not be interpreted as a mere technology investment, but as the development of processes supported by digital technologies. After the pandemic, learning models started to be discussed more in the context of planned educational actions (Hybrid, flexible education, blended learning, etc.). At the same time, the roles of teachers, students and parents, who are education stakeholders, in education have changed. Digital capacities of education systems have developed in the context of digital competencies and experiences gained after the pandemic (Sirer, 2020). It is emphasized that digital transformation requires an innovative learning model and digital learning comfort rather than just using technology in education. During the pandemic, it is seen that the development and progress of the student-centered education in the post-pandemic education programs and innovative technologies (metaverse, AR, AI etc.) are included in education (Suh and Ahn, 2022; White-Hancock, 2022).

In this research, it is aimed to determine the role of innovative technology in multicultural vocational tourism education according to student views. In line with the research purpose, the titles of multicultural education, multicultural tourism education, tourism education and digital education and innovative technologies have been examined in the literature. The limited number of studies on multicultural tourism education and innovative technologies in the literature reveals the importance of the research. To achieve the research aim, the following questions were proposed;

1. What are the effects and importance of innovative technologies on the tourism industry?
2. What are the advantages and disadvantages of vocational education in multicultural classroom?
3. What are the effects of students from different cultures on online education?
4. What are the relationship between innovative technologies and multicultural classroom in vocational tourism education?

## Method

### Research philosophy

The study was carried out using the qualitative method. Action research was used in this research. This approach was chosen because it suited the nature of the study. Since a person and a researcher with the same problem participated in the research, the action research approach was used and the researcher also served as a practitioner. Since the researcher of this research is also an educator and tries to improve the quality of the teaching process, it is designed as an action research in which the practitioner is also a researcher. While interpreting the reality of educational research, some theories emerge and there are opinions that there are gaps between these theories and practice. Teachers, one of the most important links in teaching, can fill this gap between theory and practice through action research (Johnson, 2015). Action research is the work that participants carry out in collaboration with others during the research process in order to understand, change and improve the current situation or the problems they face (Beyhan, 2013). Depending on its purpose, action research can benefit from both qualitative and quantitative research methods (Manfra, 2019). The study was carried out with the permission of the Near East University Scientific Research Ethics Committee, numbered NEU/ES/2022/905 in the 2021–22 academic years.

### Research process and study context

In this study, the researcher is also a lecturer and since it aims to increase the quality of the teaching process, the research was conducted as an action research in which the practitioner is also a researcher. Action research aims to fill research gaps by revealing the implementation process (Johnson, 2015). In this study, the purpose of the research and the data to be obtained will be used only for scientific purposes, and it was stated that participation in the research is on a voluntary basis. Feedback was received from 36 students out of a total of 50 students. For easy accessibility, interview questions were sent to the participants *via* e-mail designed with a Google Form link. After collecting the research data, it was converted into text. Afterward, the obtained data were categorized by content analysis method and themes were created. Similar statements in the data are listed. The data obtained were interpreted and concluded by tabulating.

### Working groups

The study group of this research was carried out with the participation of 36 students who received tourism education at the university in Northern Cyprus in the 2022–23 academic year. The sample was determined by the purposeful sampling method. All students in the selected class for the purpose of the research participated in the research. The demographic characteristics of the participants are as in Table 1.

**TABLE 1:** Demographic characteristics of the research participants.

	*n*	%
**Gender**		
Female	19	53
Male	17	47
**Age**		
18–24	18	50
25–29	10	28
30–34	8	22
**Nationality**		
Nigerian	15	42
Zimbabwean	13	36
North Cyprus	2	5
Zambian	2	5
Cameroonian	1	3
Jordanian	1	3
Malawian	1	3
Palestinian	1	3

The interview form created by the researcher was used as data collection tools in the research. The interview form consists of a personal information form and 11 questions. The interview form was sent to the participants online. In the study, material was collected simultaneously with the literature review to answer the research problem. The information received was analyzed and the causes of the problem were determined. In the next step, the obtained data were analyzed and the results were interpreted. The data obtained were analyzed with the content analysis method and the findings were revealed.

### Data collection tool

A “semi-structured interview form” developed by the researcher, which included instructions on how to fill out the form and personal information about the participants, was used as the data collection tool. The semi-structured interview form was presented to the opinion of two academicians and a grammar expert in terms of relevance, clarity, intelligibility, and clarity in line with the purpose of the research, and then it was given its final form. The scope and face validity of the measurement tool used with this method was ensured. Buyukozturk (2012) states that the scope and face validity of the measurement tool can be evaluated as a result of expert opinions. The research interview form consists of two parts. In the first part of the form, gender, age and nationality depending on the personal information of the researchers are included, while in the second part, the opinions and suggestions of the participants who make up the study group are included.

### Data analysis

In this study, content analysis methods were used to reveal the basic concepts and the relationships between these concepts. The basic process of content analysis is to collect similar data within the framework of certain concepts and topics, and to organize and explain them in a way that readers can understand (Yıldırım and Simsek, 2013). The codes obtained through content analysis were determined, classified and similar codes were written on the same subject. Topics are categorized according to general meanings. Finally, categories were given according to each question and the categories were quantitatively indicated by frequency.

For the validity of the study, the data collection process was followed by the researcher and the data obtained from the participants were examined and arranged by the researcher. After the arrangement, the themes and codes were determined by considering the literature review made before the interviews (Yıldırım and Simsek, 2013).

For the reliability of the research, opinions were taken from two academicians and adjustments were made where necessary so that the data under the themes could form a meaningful whole (internal consistency) and then form a whole among them (external consistency). In addition, in order to ensure the reliability of the study, expert opinions were consulted, confirming whether the opinions given under the six headings reached in the study represent the issues discussed. By comparing the evaluations of the views to be included in the themes by the researcher and the expert, the numbers of “consensus” and “disagreement” were determined, the formula put forward by Miles and Huberman (1994) was used and the reliability of the research was calculated. A result of 80% or more as a result of the calculation is accepted as an indication of the required reliability (Miles and Huberman, 1994). 92% reliability was achieved in the reliability study for this study.

## Findings

The findings obtained in line with the opinions of 36 students participating in the research were analyzed. In addition to the research questions, participants were asked whether being in multicultural classrooms provided them with a different perspective. While 86% of the participants answered yes, 14% answered no. This finding shows that the majority of the participants believe that the multicultural class provides them with different perspectives. The data obtained in line with the research questions are as shown in Table 2.

**TABLE 2:** Research findings according to participants’ view.

Category	Themes		Frequency *N*	Percent %
The effects and importance of innovative technologies on the tourism industry	Provides easier access to target market		23	41
	It enables the market to grow		11	20
	Facilitates data analysis		8	14
	have significant impact		5	9
	Meets the expectations of tourists and provides satisfaction		5	9
	Makes traveling easier		4	7
	Total view		56	100
Advantages and disadvantages of vocational education in multicultural classroom	Advantages	Getting to know different cultures	18	37
		Provides social awareness	7	14
		Making friends in different cultures	7	14
		Encourage tolerance	6	12
		Raising awareness	5	10
		Developing a positive attitude	3	6
		Desire to learn a different language	3	6
	Total view		49	100
	Disadvantages	Prejudice	14	29
		Language barrier	12	25
		Culture clash	7	15
		Racism	6	13
		Communication problem	5	10
		Estrangement	4	8
	Total view		48	100
Effects of students from different cultures on online education	Positive	Getting to know and discussing different cultures	17	32
		Different perceptions	15	29
		Different educational technologies enable effective learning	7	13
		Developing technological skills	5	10
		Use of different communication tools	4	8
		Equal educational opportunity	4	8
	Total view		52	100
	Negative	Language barrier	21	46
		Prejudice	10	22
		Lack of application	6	13
		Compatibility problem	5	11
		Poor academic performance	3	7
		Time differences	1	2
	Total view		46	100
Innovative technologies and multicultural classroom in vocational tourism education	Improves learning process and strategies		23	41
	Supports effective learning		9	16
	Provides opportunity to share knowledge and experience		7	13
	Enables new education model		5	9
	Offers practical alternatives		5	9
	The problem is solution oriented.		4	7
	Strengthens communication		3	5
	Total view		56	100

The opinions of the participants on the impact and importance of innovative technologies in the tourism industry are as follows.

*“Tourists can be sure that they will receive better defined services, which will significantly increase their satisfaction. The main objective of implementing tourism technology is to improve service provision and thereby provide tourists with value for their hard-earned money.”* S4

*“Using data analytics, customers can make more informed choices with the help of virtual travel assistants. In addition, artificial intelligence is used in some digital applications to automate repetitive tasks, and Chabot systems using machine learning can help customers.”* S11

*“It helps to narrow down and meet your target audience. It gives you a broader view of the sector. It aids soothe transaction between various countries, globalization, International trade.”* S28

The students participating in the research mostly defined the effects and importance of innovative technologies on the tourism industry as facilitating access to the target market. They emphasized that the impact of innovative technologies is important and besides enabling the market to grow, they also have effects on facilitating travel, meeting the expectations of tourists and ensuring tourist satisfaction. Sönmez (2020) states that innovation technologies are important in the development of the tourism industry, providing competitive advantage and customer satisfaction. Research shows that innovation technologies have an important role in the tourism sector and facilitate the growth and development of the market (Alderbert et al., 2010; Omerzel, 2015).

Students’ views on the advantages and disadvantages of vocational education in multicultural classrooms were examined under two themes, advantages and disadvantages. Knowing different cultures was the most mentioned advantage. Prejudice and language barrier are the most emphasized disadvantages. Many studies investigating the problems experienced in multicultural education have put forward the issues of language, harmony and prejudice as problems that need to be resolved (Leung and Hue, 2020; Portera, 2020; Vilog, 2021; Roberson, 2022). The opinions of the participants are as follows.

*“It enables students to acquire the skills to interact with people from different cultures, communicate and promote tolerance and develop a positive attitude in order to create a moral and civic society. An important problem of multiculturalism is the language barrier.”* S8

*“Students frequently have excellent social awareness and are better prepared to function in varied classroom or job environments. Disadvantages; It is possible that teachers will find it challenging to assess how well their students are comprehending the subject matter. There is a language barrier created by the fact that not all students come from the same background.”* S15

*“The advantages are it allows others to know about other culture, food, history and how they behave generally. The disadvantages are language barriers and misunderstandings in communication and behavior.”* S31

The effects of online education on students in multicultural classrooms were examined. The effects revealed were analyzed as positive and negative effects. As a positive effect, the participants expressed their views of getting to know different cultures and having a discussion environment, as well as acquiring different perspectives. In addition, different educational technologies provide effective learning, provide equal educational opportunities, use different communication tools, different perspectives and development of technological skills are among the positive opinions. The negative opinion expressed by the majority is the language barrier. Prejudice, lack of practice, adjustment problems, poor academic performance and time differences were expressed as other negative aspects. The statements made by the participant students are as follows.

*“The study also found that online educators should design courses to address potential cultural barriers such as language, media use, and plagiarism.”* S9

*“Positive effects: students get to see different sets of people from the country and different cultures. Negative: Students sometimes find it add to adjust to other people’s culture.”* S23

*“In my opinion, there is more of a positive effect because we can learn and understand how people from all over the world view a topic and increase knowledge using these experiences. Lack of practical is negative effects”* S35

In international distance education, each student’s cultural experiences, cultural identities, and the level of development of multicultural counseling and social justice competencies may differ (Chen et al., 2020). Therefore, it is predicted that the use of innovative technology in multicultural classrooms will enable students to get involved on a common ground.

Students’ opinions on the interaction between innovative technologies and multicultural classes in vocational tourism education were taken. The answers of the participants were gathered under seven themes. When the participant answers were examined, many participants emphasized that it was effective in improving the learning process and strategies. The following are the participant opinions regarding the research findings.

*“For example cloud applications, allow us to save, share and access information on any device with an internet connection as well as user’ computers. In education, digital textbooks, lesson plans, videos and assignments can be stored and shared in the cloud. It is also used to allow them to chat with teachers and other classmates. These and similar technologies provide students with a new educational model and strategies such as improving their discussion, research, group work and analysis skills.”* S7

*“The use of technology in education allows teachers to improve their teaching strategies and learning processes and to make all kinds of learning styles more comprehensive. Thanks to technology, the world is getting smaller by sharing knowledge and experiences. It is possible to see this in today’s multicultural and multilingual classrooms.”* S26

*“Innovative technologies have really been one of the solutions to the problems faced in multicultural classrooms in terms of vocational education, so I will say that it is really helpful and impactful.”* S33

## Conclusion and discussion

In line with the results obtained in the research, the participants stated that they provided positive opinions about the importance and effects of innovative technologies in the tourism sector and provided the growth of the market by facilitating access to the target market. Many studies reveal that innovative technologies provide significant advantages for tourism marketing (Dredge et al., 2019; Pencarelli, 2020). Revealing the impact and importance of innovative technologies in the tourism industry reveals the awareness of students studying in multicultural education environments. Therefore, the advantages and disadvantages of multicultural vocational education were examined. While the prominent advantage knows different cultures, the disadvantage is determined as prejudice and language barrier. Many studies on multicultural education in the literature reveal similar advantages and disadvantages with research results (Benediktsson and Ragnarsdottir, 2019; Guclu Yılmaz, 2020; Munalim, 2020). Positive and negative views of the multicultural education environment in online education were asked, and in the same way, knowing different cultures and having different perspectives were revealed as positive effects, but language problem and prejudice were revealed as the most emphasized negative effects (Uyun and Warsah, 2022). Finally, the views of the participants on innovative technologies and multicultural education in vocational tourism were examined. Participating students mostly stated that they provided improvement of the learning process and strategies.

This study examines the role of innovative technology in multicultural vocational tourism education in line with students’ views. In this context, important data are provided to the literature. In the tourism industry, which offers many international job opportunities, the positive and negative views of students on multicultural education are examined, it is seen that there is not enough research on the use of innovative technologies in vocational tourism education. This study contributes to the literature in this respect. In addition, the findings obtained from the data can contribute to universities and authorized institutions in the creation of innovative technology supported tourism education programs for international students. The research is limited to the study group. Since this study was designed as a qualitative study, the results cannot be generalized and are limited to the answers given by the participant students’ own experiences and opinions. It is recommended to continue student-centered education after the pandemic, especially to provide innovative technology infrastructures of vocational education programs and to increase the qualifications of lecturers in this direction. In future studies, the opinions of lecturers in this direction can be examined and the results of the study can be compared.

## Data availability statement

The datasets presented in this study can be found in online repositories. The names of the repository/repositories and accession number(s) can be found below: https://osf.io/snw6t/.

## Ethics statement

Written informed consent was obtained from the individual(s) for the publication of any potentially identifiable images or data included in this article.

## Author contributions

NB was responsible for designing study, collecting data, analyzing data, and the writing of the article and approved the submitted version.
